# Supplementary material for: Life cycle net energy assessment of sustainable H2 production and hydrogenation of chemicals in a coupled photoelectrochemical device
Source: Nat Commun. 2023 Feb 22;14:991. doi: 10.1038/s41467-023-36574-1 (PMC9947173; doi:10.1038/s41467-023-36574-1)
Supplement: Supplementary file 1 — Supplementary Information [file 41467_2023_36574_MOESM1_ESM.pdf]

Supplementary Information

Life cycle net energy assessment of sustainable H_2 production and hydrogenation of chemicals in a coupled photoelectrochemical device

Xinyi Zhang[1,2], Michael Schwarze[2], Reinhard Schomäcker[2], Roel van de Krol[1,2], Fatwa F. Abdi[1,*]

[1]Institute for Solar Fuels, Helmholtz-Zentrum Berlin für Materialien und Energie GmbH, Hahn-Meitner-Platz 1, 14109 Berlin, Germany

[2]Technische Universität Berlin, Department of Chemistry, TC8, Straße des 17. Juni 124, 10623 Berlin, Germany

*correspondence: fatwa.abdi@helmholtz-berlin.de

Supplementary Note 1. Estimation of the market size of MSA

Due to the scarcity of market data on the production or consumption of methyl succinic acid (MSA), we performed an estimation of the market size based on the data on itaconic acid (IA) as the feedstock and a proxy chemical, succinic acid (SA).

The US department of Energy identified IA as one of the twelve building blocks that possess the potential to be transformed subsequently to a number of high-value bio-based chemicals or materials.[1] IA has been commercially produced biochemically by fermentation mainly using *Aspergillus terreus* with a significant production yield.[2] The global production of the IA in 2011 was 41,400 t and was projected to increase to 407,790 t in 2020 with $567.4 million market value.[3] One of the valuable chemical compounds that can be produced from IA is MSA, whose derivatives are ubiquitously used as solvents in cosmetics,[4] polymer synthesis,[5] binders in powder coatings,[6] and in organic synthesis especially for pharmaceutical synthesis.[7,8] Because of the high manufacturing cost as well as the fact that only a few end-use applications with small volume markets have been identified and not developed yet, the current niche market of MSA is limited, and there is no market report available. Currently, the hydrogenation of IA is the only method to produce MSA at small scale based on customized orders.[9-13] This conventional hydrogenation process usually applies typical catalysts, such as Raney Ni, Pd/C and Ru/C, at 25-150 °C and 1-140 bar H_2.[14] Therefore, we made a preliminary deduction of the global MSA production based on the IA market; 3.7% of the annual production of IA (15,088 t) is consumed for subsequent chemical synthesis.[3] Assuming that this goes into the hydrogenation process to produce MSA, this would lead to a total annual MSA production of 15,322 t.

We also investigated SA which has similar chemical features as MSA for a further justification of our estimation. SA is a popular chemical intermediate that is produced in large-scale through catalytic hydrogenation,[15] with a total addressable market of 40,000 t in 2011 and 699,499 t in 2020 as projected.[3] Although MSA does not occupy as large market as SA due to its high cost and early-stage development,

the potential application of PEC-produced MSA as a green chemical is expected to decrease the cost of MSA production and increase its market size.

Table S1. Component parameters and processes considered for the coupled PEC hydrogen production and hydrogenation of IA to MSA.

Component	Material	Process[a]	Thickness / Parameter	Data source
Bottom absorber	SHJ cell	–	180 μm	Ecoinvent[b], literature [16]
Top absorber	$BiVO_4$	spray pyrolysis	200 nm	Laboratory data[c], Ecoinvent, literature [17]
Hydrogen evolution catalysts	Pt on 2 mm glass	electrodeposition	20 nm	Ecoinvent, literature [18]
Oxygen evolution co-catalysts	Co-P_i	electrodeposition	30 nm	Ecoinvent, literature [18]
Device cover	Solar grade glass	–	5 mm	Ecoinvent, literature [18]
Device chamber	Polyvinyl chloride (PVC)	–	5 mm	Ecoinvent, literature [18]
Membrane	Nafion	–	50 μm	Ecoinvent, literature [18]
Intervening layer	PEDOT-PSS	–	20 μm	Ecoinvent, literature [18]
Homogenous catalyst	Rhodium TPPTS	–	2.5 mmol/m^2 (0.64 mM)	Ecoinvent, Laboratory data

[a] The required fabrication steps that consume energy are considered. Empty fields refer to the case in which only manual assembly with negligible energy demand is required and/or the energy demand of the process is already included in the data obtained from the database.

[b] The Ecoinvent data of the SHJ cell were adjusted for single-sided metallization.

[c] Energy and material consumption were estimated from our laboratory experimental data. 50% thermodynamic efficiency of the fabrication process was assumed for the calculation.

Supplementary Note 2. Uncertainty analysis for the device-level study considering three different scenarios (lower, base and higher cases)

Since the coupled PEC concept is at a very early stage of research at the device-level, the life cycle inventory data used during its fabrication and operation has unavoidable uncertainties. This uncertainty analysis addresses this limitation by including variations in material choices and fabrication parameters under three assumed cases as shown in in Table S2. The same top and bottom absorbers were used in all cases since this corresponds to our research target and the specific preliminary demonstration device in our lab. Other material choices were made based on their respective primary energy demands and categorized into lower and higher cases. For the cumulative energy demand of fabrication process, uncertainty was introduced to the energy usage based on different assumptions of thermal and electricity conversion efficiency.

Table S2. Assumptions for lower, base, and higher cases of uncertainty analysis at the device-level.

Category	Component	Lower case	Base case	Higher case
Material choices	Bottom absorber	SHJ cell	SHJ cell	SHJ cell
	Top absorber	$BiVO_4$	$BiVO_4$	$BiVO_4$
	Photocathode catalyst	Cobalt	Platinum	Platinum
	Photoanode catalyst	No catalyst	Co-Pi	Iridium (Ir)
	Chamber	3 mm PVC	5 mm PVC	5 mm Polycarbonate
	Membrane	30 μm Nafion	50 μm Nafion	70 μm Nafion
	Homogenous catalyst	Nickel	Rh-TPPTS	Pd/C
Fabrication	Thermal efficiency	30%	50%	70%
	Electricity conversion efficiency	0.27	0.29	0.33

Supplementary Note 3. Preliminary system-level analysis for a 100 m^2 coupled PEC system

To investigate the additional energy demand from factors beyond the PEC device itself (e.g., balance-of-system), a preliminary system-level analysis was also conducted. We considered and adapted the technical and engineering designs of a recently reported 100 m^2 photochemical water splitting system in Japan.[27] A single PEC device has a dimension of 295 mm × 250 mm and 625 cm^2 photoactive area. One panel consists of 48 devices, and the overall 100 m^2 system consists of $33\frac{1}{3}$ panels of 3 m^2 each. The total light absorbing area is 70 m^2, while the remaining 30 m^2 of land area is attributed for row spacing.

De-ionized water is used for the electrolytes (as also the case in our laboratory experiment), and the large amount of water feedstock is assumed to be treated on-site using grid water with reverse osmosis (RO) process.[28] The energy required for water pumping from the distribution station to the prospective system is calculated based on the average water transportation consumption. The energy demand depends on the distance and usage of water while the specific energy use for water pumping is based on the report of Plappally and Lienhard.[29] The average water delivery distance is assumed to be 10 km, and the daily water consumption is calculated based on the day in which peak generation occurs within the year, i.e., summer solstice (7.17 kWh/m^2/day).

Gas handling consists of blower, dryer, and compressor which pressurizes H_2 to 300 psi at temperature of 20 °C for delivery to end-users through pipelines. Sathre *et al* reported in their LCA of large-scale PEC system that the energy demand for gas handling accounts for 6.7% of the total primary energy investment and 34.7% of annual operational energy demand.[30] The same considerations are used in our study.

After the coupled PEC hydrogenation reaction takes place in the PEC device at a particular H_2-to-MSA conversion efficiency, the catholyte contains a mixture of IA, Rh-TPPTS, H_2 gas and MSA, which is delivered to a separating unit. Compressed H_2 gas is collected and delivered to pipelines, and the remaining liquid solution is sent to another separating unit for MSA extraction. Recent separation techniques, such as

micellar-enhanced ultrafiltration and cloud point extraction,[31,32] have been reported for the separation of IA, MSA, and the homogenous catalyst. Conventionally, the catalyst is filtered off and suitable acids (hydrochloric acid, sulfuric acid) is added to extract MSA from its metal salt.[9] However, these processes are not yet commercialized, and for our system-level analysis we consider the separation process of succinic acid as a proxy process for MSA separation.[33] As reported, the downstream separation and purification contribute to 16% of the total cost in the SA production line. The same ratio was taken for the energy demand of MSA separation in our study to estimate the CED of this process.

The replacement interval of the device components is assumed to be once per year and 10% of device components is replaced.[30] At the end of the system service time, 10% capital energy is required for decommission.[34]

Uncertainties were introduced to the fixed and O&M components of the system, according to cases listed in Table S3. As a regional relevant uncertainty, different solar intensities across Germany (Hamburg, German average, and Munich) were used for the lower, base, and higher cases.

Table S3. Assumptions for lower, base, and higher cases of uncertainty analysis at the system level.

System components	Unit	Lower case	Base case	Higher case
Fixed energy demand				
PEC device	MJ/m^2	3,434	3,834	4,462
Gas handling	% of capital demand	4.7%	6.7%	11.7%
Decommission	% of capital demand	5%	10%	15%
Separation unit	% of capital demand	10%	16%	20%
O&M energy demand				
Annual replacement	% of PEC device	5%	10%	15%
CED of IA feedstock	MJ/kg	5.9	8.4	10.9
Water usage	$kg/day/m^2$	0.16	0.18	0.20
Water delivering	$kWh/m^3 \cdot km$	0.002	0.005	0.007
Gas handling	% of H_2 production	29%	35%	36%
MSA separation	% of coupled process	10%	16%	20%
Solar intensity	$kWh/m^2/day$	2.9	3.5	3.7

Table S4. Cumulative energy demand (CED) of inputs parameters for preliminary system-level analysis for a 100 m^2 coupled PEC system. The error bars correspond to the uncertainty introduced in Table S3.

System components	Energy demand (MJ/m^2)		
	Lower case	Base case	Higher case
Fixed energy demand			
PEC device	3,434	3,834	4,462
Gas handling	160.8	257.3	522.4
Decommission	179.7	409.1	747.7
Separation unit	359.5	651.7	996.9
O&M energy demand			
Replacement	3,434	3,834	4,462
IA usage	3,425.7	4,076.8	4,361.4
Water usage	3.1	3.7	4.0
Water delivering	0.03	0.07	0.13
Gas handling	0.9	1.3	1.5
MSA separation	686.4	1,260.9	1,765.8

Table S5. Cumulative energy demand (CED) for H_2 production with different methods.

Technology	CED (MJ/kg H_2)
Wind/electrolysis [19]	9.1
Natural gas steam reforming [20]	183.2
PV-electrolysis[21]	187.5
Country grid mix/electrolysis [22]	192 - 217.8
PEC water splitting (microwire Si) [18]	10 - 194
PEC water splitting (SHJ/$BiVO_4$; this study)	34 - 680

Table S6. Potential alternative materials for substituting major energy-consuming components (bottom absorber and membrane) in our PEC cell.

Alternatives	Primary energy demand (MJ/m^2)
Bottom absorber candidates	
Perovskite-Perovskite (flexible substrate) [23]	779
Perovskite-Perovskite(glass) [23]	823
Planar-structured Si [23]	860
SHJ cell (This study)	2,530
Perovskite-Si [23]	3,723
Membrane candidates	
Nafion	139
Polysulfone [24]	12.2
Porous polybenzimidazole (PBI) [25]	26.3
Silica [25]	298

Table S7. For materials that are unavailable in Simapro for the modeling of PEC devices, representative data on proxy materials are used.[26]

**Material**	**Proxy material**
Bismuth	Tellurium
Vanadium	Tellurium
Cobalt	Nickel

Table S8. Life Cycle Inventory data for the silicon heterojunction (SHJ) solar cell used as the bottom absorber in the coupled PEC device.

Process input	Unit (per m^2 cell area)	Amount
Wet etching: Texturing/cleaning		
Water (deionized)	L	33.43
Electricity	kWh	0.65
Hydrogen fluoride	kg	0.10
Sodium fluoride	kg	0.16
Hydrogen Peroxide	kg	0.06
Hydrogen acid	kg	0.06
Ammonia	kg	0.01
Compressed air	m^3	0.25
Fluid waste to treatment	L	33.50
Thin-film deposition: PECVD of a-Si:H		
Electricity	kWh	6.59
Water	L	394.00
Silane	g	1.62
Hydrogen	g	2.42
Oxygen	g	0.26
Boron trifluoride	g	2.20
Nitrogen trifluoride for cleaning	g	29.00
Gaseous waste to abatement	L	
TCO Sputtering		0.52
Electricity	kWh	1.10
Water	L	29.60
ITO	g	
Metallization (single side)		
Screen printing on backside		0.52
Electricity	kWh	1.10
Compressed air	m^3	29.60
Silver paste	g	33.43

Table S9. Life Cycle Inventory data for the $BiVO_4$ photoelectrode and catalysts deposition. Electricity use for heating and deposition processes was calculated with a thermo-efficiency of 50%.

Process input	Unit (per m^2 cell area)	Amount
Spray pyrolysis of $BiVO_4$		
$Bi(NO_3)_3.5H_2O$	g	0.02
acetic acid	mL	8.75
$VO(AcAc)_2$	g	0.01
absolute ethanol	mL	6.58
Electricity for heating	kWh	0.03
Nitrogen gas	Mg	0.74
Electricity for valve	kWh	4.31
Electrodeposition of Co-P_i catalyst		
Co-P_i	g	0.10
Electricity	kWh	1.88
Electrodeposition of Platinum catalyst		
Platinum	g	0.03
Solar grade glass	kg	5.00
Electron beam deposition of Pt		
Electricity	kWh	1.21

Table S10. Life Cycle Inventory data for the membrane, encapsulation, and other ancillary processes considered in our coupled PEC device.

**Process input**	**Unit (per m^2 cell area)**	**Amount**
Membrane Fabrication		
Nafion	g	79
Encapsulation		
flat glass	kg	7.5
polyvinyl chloride (PVC)	kg	6.9
Other ancillary processes		
miscellaneous chemicals	MJ	15
water pumping	MJ	31
cleaning	MJ	10
environmental control	MJ	200
water treatment	MJ	0.001
water consumption	kg	0.05

Table S11. Life Cycle Inventory data for methyl succinic acid (MSA) production through conventional hydrogenation[5] and fermentation approaches.[12]

Process input	Unit (per kg MSA)	Amount
Hydrogenation method		
Itaconic acid	kg	0.99
Methanol	mL	79.97
Nickel catalyst	g	197.44
Hydrogen gas	g	0.14
Electricity	kWh	3.92
Fermentation method		
glucose	g	2,986.90
KH_2PO_4	g	13.28
NH_4NO_3	g	49.78
$MgSO4.7H_2O$	g	16.59
$CaCl_2.2H_2O$	g	82.97
$FeCl_3.6H_2O$	g	0.03
$ZnSO_4.7H_2O$	g	0.13
$CuSO_4.5H_2O$	g	0.25
Itaconic acid	g	984.85
Sulfuric acid (0.5M)	mL	13,275.13
Electricity	kWh	614.80

Supplementary Note 4. Estimation of N_2 consumption

The Weymouth equation[35,36] was used to estimate the N_2 gas consumption in the fabrication process of $BiVO_4$:

$$Q = 433.5 \times (\frac{T_{\mathrm{SC}}}{P_{\mathrm{SC}}}) \times (\frac{{P_1}^2 - e^s {P_2}^2}{G\, L_e T_{\mathrm{avg}} Z})^{0.5} \times D^{2.667} \times E \quad \text{(S1)}$$

$$s = \frac{0.0375 G \times \Delta z}{T_{\mathrm{avg}}} \quad \text{(S2)}$$

$$L_e = \frac{(e^s - 1) \times L}{s} \quad \text{(S3)}$$

T_{SC} and P_{SC} are the temperature and pressure at standard conditions, T_{avg} is the average temperature of the gas line, P_1 and P_2 are the pressures at the pipe entrance and exit in absolute psi, L is the length of the pipe in miles, G is the relative gas density with respect to air, Z is the gas compressibility, E is the pipeline efficiency, L_e is the effective length of the pipeline, Δz is the elevation of the pipe exit with respect to the entrance in feet, and Q is the flow rate in standard cubic feet per day.

The gas compressibility Z and density were calculated at the average pressure (P_{avg}) and temperature, as defined below:

$$P_{\mathrm{avg}} = \frac{2}{3} \times (\frac{{P_1}^3 - {P_2}^3}{{P_1}^2 - {P_2}^2}) \quad \text{(S4)}$$

$$T_{\mathrm{avg}} = \frac{T_2 - T_1}{\ln(\frac{T_1 - T_g}{T_2 - T_g})} + T_g \quad \text{(S5)}$$

T_1 and T_2 are the temperatures before and after the gas are compressed. T_g is the critical temperature of the medium gas.

Supplementary Note 5. Uncertainty analysis for methyl succinic acid (MSA) production using conventional method

The input mass and energy data into MSA production is based on information found in the literature, which was used to determine the cumulative energy demand (CED) of conventional hydrogenation method of producing MSA. Considering the immaturity of manufacturing techniques and the fluctuation of operating conditions of MSA production, three main energy contributors were selected to address this inherent uncertainty: electricity consumption, hydrogenation catalysts and itaconic acid (IA) feedstock. For electricity consumption of the production equipment, the thermal efficiency was assumed to be 30%, 50% and 70%.[18] The hydrogenation of IA to MSA has been reported with several hydrogenation catalysts, such as Raney Ni, Pd/C, Ru/C etc.[11,13,37] Iron-based catalysts are also expected to be developed for such a process, and this was considered for our analysis due to its low energy demand.[26] As for the IA feedstock, Nieder-Heitmann *et al* has reported a 30% uncertainty in their life cycle analysis.[38] Table S12 shows a summary of the input parameters and the percentage of uncertainty being introduced by each parameter to the individual and total MSA production process. The range of inputs uncertainty is shown in Figure S1 with errors bars indicating the upper and lower limits of the energy demand.

Table S12. Life Cycle cumulative energy demand (CED) of inputs parameters in unit of MJ/kg MSA production and output percentage of uncertainty of MSA production

**Input parameters**	**Cumulative energy demand (MJ/kg MSA) and uncertainty**		
	Low	Base case	High
**Itaconic acid**	5.83	8.33	10.83
**Catalyst**	4.60	31.10	161.87
**Electricity**	25.32	42.20	59.08
**Total**	64.80	84.18	234.32

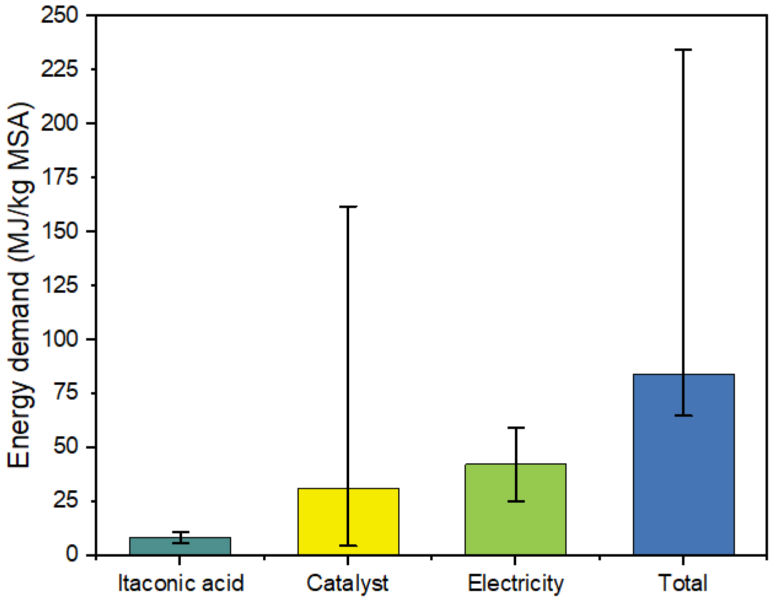

Figure S1. The CED of MSA inputs parameters with error bars showing the range of uncertainties based on cases described in Table S12.

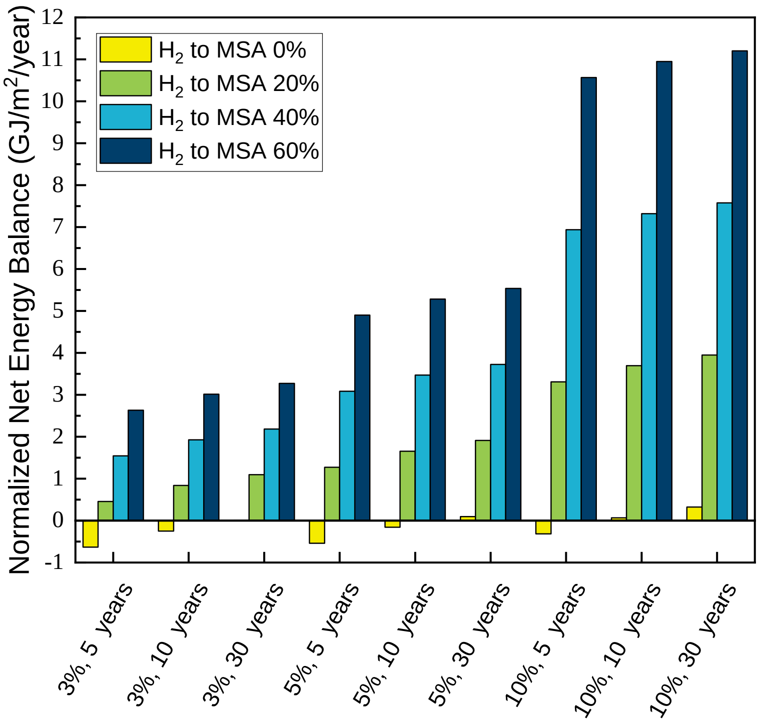

Figure S2. The normalized net energy balance for various STH efficiencies, and device longevities when applying different levels of H_2-to-MSA conversion from 0% to 60%.

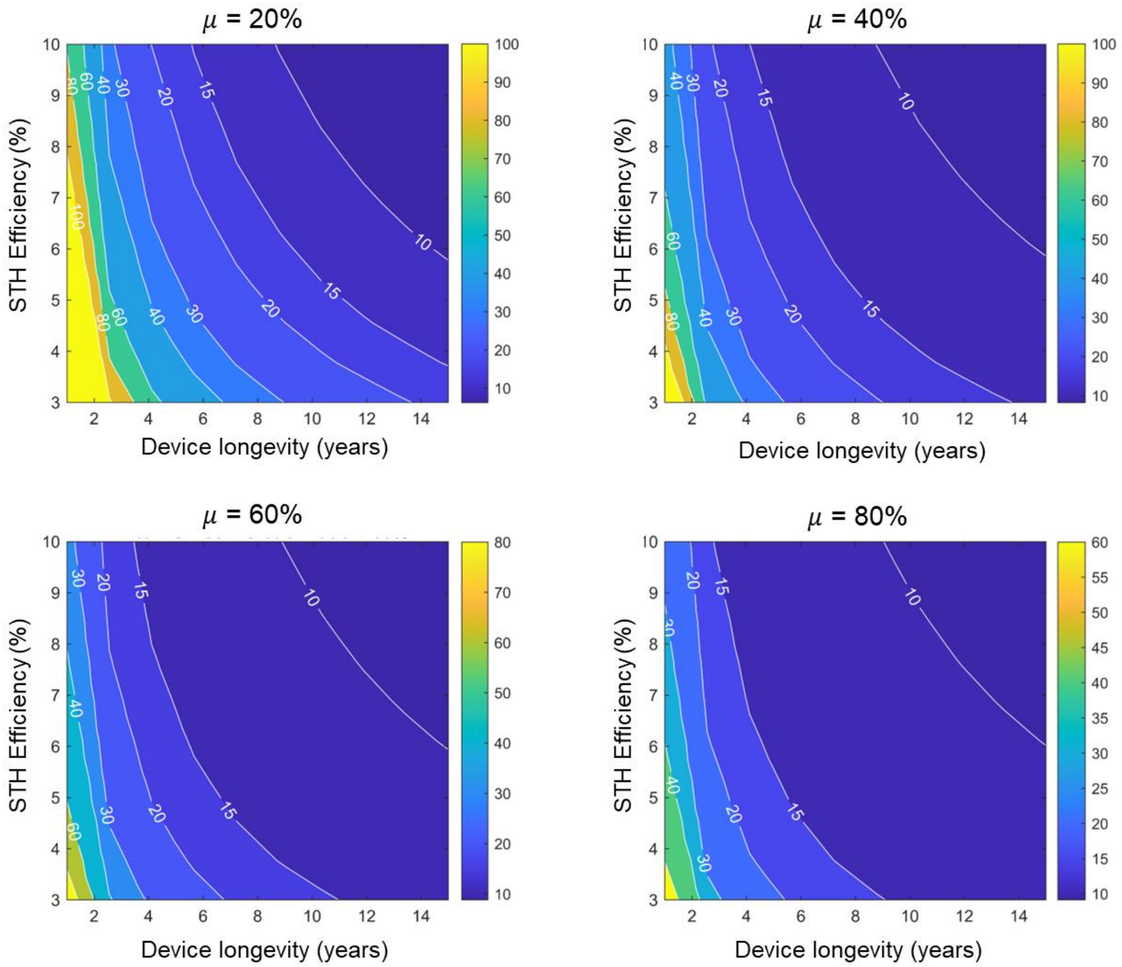

Figure S3. The cumulative energy demand (MJ) to produce 1 kg of MSA when the H_2-to-MSA conversion efficiency (μ) ranges from 20% to 80% for various STH efficiencies and device longevities.

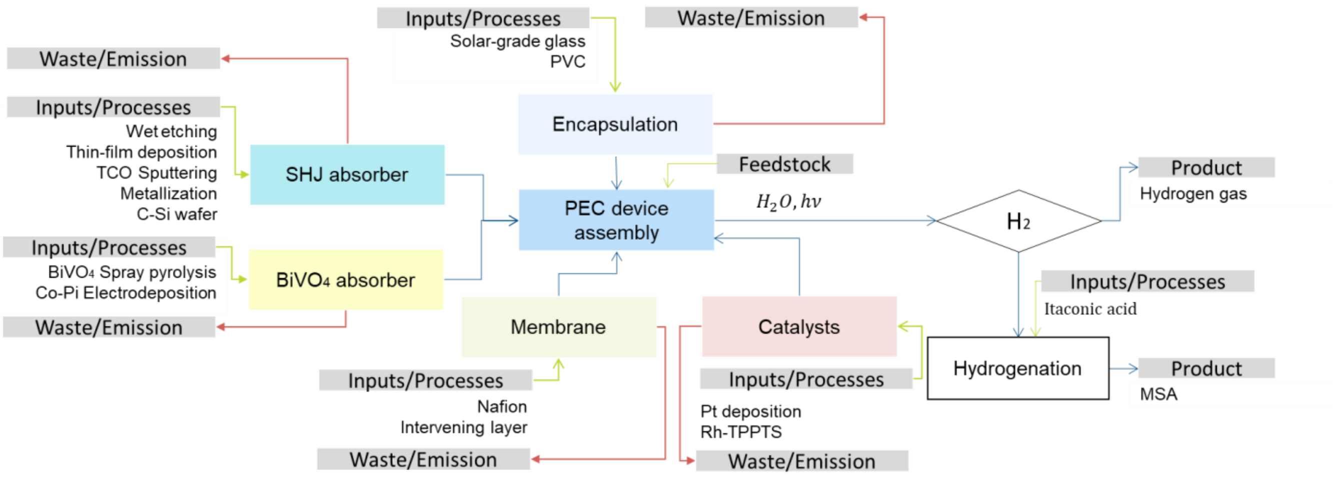

Figure S4. Generic process flow diagram (PFD) with the primary level processing used in the Simapro calculation setup.

Supplementary References

1 Werpy, T. & Petersen, G. Top value added chemicals from biomass: volume I--results of screening for potential candidates from sugars and synthesis gas. (National Renewable Energy Lab., Golden, CO (US), 2004).

2 Kautola, H., Vassilev, N. & Linko, Y.-Y. Itaconic acid production by immobilized Aspergillus terreus on sucrose medium. *Biotechnol. Lett.* **11**, 313-318 (1989).

3 Weastra, S. Determination of market potential for selected platform chemicals—itaconic acid, succinic adis, 2, 5-furandicarboxylic acid. *Bratislava, Slovakia* (2016).

4 Richard, H. & Muller, B. Use of a 2-methylsuccinic acid diester derivative as solvent in cosmetic compositions; cosmetic compositions containing the same. WO2012119861A3 (2012).

5 Verduyckt, J. & De Vos, D. Method for the production of methylsuccinic acid and the anhydride thereof from citric acid. WO2018065475A1 (2017).

6 Mijolovic, D., Szarka, Z. J., Heimann, J. & Garnier, S. Powder coating useful as a coating agent, and for coating metallic- and non-metallic surfaces, comprises a binder comprising methyl succinic acid. DE102011080722A1 (2012).

7 Okabe, M., Lies, D., Kanamasa, S. & Park, E. Y. Biotechnological production of itaconic acid and its biosynthesis in Aspergillus terreus. *Appl. Microbiol. Biotechnol.* **84**, 597-606 (2009).

8 Willke, T. & Vorlop, K.-D. Biotechnological production of itaconic acid. *Appl. Microbiol. Biotechnol.* **56**, 289-295 (2001).

9 Abraham Bavley & J, K. C. Preparation of methylsuccinic acid and metal salts thereof. The USA patent US-2773897-A (1956).

10 Shi Huancong, S. W., Xu Hualong. Catalytic synthesis process of methyl succinic acid. China patent CN 1609089 (2005).

11 Wu Yumin, G. C., Ren Yinyin, Wang Chuanxing. Xu Jun. Preparation method for 2-methylsuccnic acid through Ni catalysis. China patent CN 102617326 (2012).

12 Holzhäuser, F. J. *et al.* Electrocatalytic upgrading of itaconic acid to methylsuccinic acid using fermentation broth as a substrate solution. *Green Chem.* **19**, 2390-2397 (2017).

13 Huang, Q. *et al.* Preparing acid-resistant Ru-based catalysts by carbothermal reduction for hydrogenation of itaconic acid. *RSC Adv.* **5**, 97256-97263 (2015).

14 Verduyckt, J. & De Vos, D. E. Highly selective one-step dehydration, decarboxylation and hydrogenation of citric acid to methylsuccinic acid. *Chem. Sci.* **8**, 2616-2620 (2017).

15 Saxena, R., Saran, S., Isar, J. & Kaushik, R. in *Current developments in biotechnology and bioengineering* 601-630 (Elsevier, 2017).

16 Louwen, A., van Sark, W. G. J. H. M., Schropp, R. E. I., Turkenburg, W. C. & Faaij, A. P. C. Life-cycle greenhouse gas emissions and energy payback time of current and prospective silicon heterojunction solar cell designs. *Prog. Photovolt.: Res. Appl.* **23**, 1406-1428, doi:https://doi.org/10.1002/pip.2540 (2015).

17 Ahmet, I. Y. *et al.* Demonstration of a 50 cm^2 $BiVO_4$ tandem photoelectrochemical-photovoltaic water splitting device. *Sustain. Energy Fuels* **3**, 2366-2379, doi:10.1039/C9SE00246D (2019).

18 Zhai, P. *et al.* Net primary energy balance of a solar-driven photoelectrochemical water-splitting device. *Energy Environ. Sci.* **6**, 2380-2389, doi:10.1039/C3EE40880A (2013).

19 Mann, M. & Spath, P. Life cycle assessment of renewable hydrogen production via wind/electrolysis: Milestone completion report. (National Renewable Energy Lab., Golden, CO.(US), 2004).

20 Spath, P. L. & Mann, M. K. Life cycle assessment of hydrogen production via natural gas steam reforming. (National Renewable Energy Lab.(NREL), Golden, CO (United States), 2000).

21 Granovskii, M., Dincer, I. & Rosen, M. A. Exergetic life cycle assessment of hydrogen production from renewables. *J. Power Sources* **167**, 461-471 (2007).

22 Ivy, J. Summary of electrolytic hydrogen production: milestone completion report. (National Renewable Energy Lab., Golden, CO (US), 2004).

23 Tian, X., Stranks, S. D. & You, F. Life cycle energy use and environmental implications of high-performance perovskite tandem solar cells. *Sci. Adv.* **6**, eabb0055, doi:doi:10.1126/sciadv.abb0055 (2020).

24 Wernet, G. *et al.* The ecoinvent database version 3 (part I): overview and methodology. *Int. J. Life Cycle Assess.* **21**, 1218-1230, doi:10.1007/s11367-016-1087-8 (2016).

25 Navajas, A. *et al.* Environmental Evaluation of the Improvements for Industrial Scaling of Zeolite Membrane Manufacturing by Life Cycle Assessment. *ACS Sustain. Chem. Eng.* **6**, 15773-15780, doi:10.1021/acssuschemeng.8b04336 (2018).

26 Nuss, P. & Eckelman, M. J. Life cycle assessment of metals: a scientific synthesis. *PloS one* **9**, e101298 (2014).

27 Nishiyama, H. *et al.* Photocatalytic solar hydrogen production from water on a 100-m^2 scale. *Nature* **598**, 304-307, doi:10.1038/s41586-021-03907-3 (2021).

28 Hayter, S., Tanner, S., Urbatsch, E. & Zuboy, J. Saving energy, water, and money with efficient water treatment technologies. *US Department of Energy, National Renewable Energy Laboratory (NREL), Federal Energy Managment Program (FEMP)* (2004).

29 Plappally, A. Energy requirements for water production, treatment, end use, reclamation, and disposal. *Renewable Sustainable Energy Rev.* **16**, 4818-4848 (2012).

30 Sathre, R. *et al.* Life-cycle net energy assessment of large-scale hydrogen production via photoelectrochemical water splitting. *Energy Environ. Sci.* **7**, 3264-3278 (2014).

31 Schwarze, M. Recycling of Catalysts from Surfactant Systems. *Chem. Ing. Tech.* **93**, 31-41 (2021).

32 Schmidt, M. *et al.* Hydrogenation of Itaconic Acid in Micellar Solutions: Catalyst Recycling with Cloud Point Extraction? *Ind. Eng. Chem. Res.* **58**, 2445-2453 (2018).

33 Efe, Ç., van der Wielen, L. A. & Straathof, A. J. Techno-economic analysis of succinic acid production using adsorption from fermentation medium. *Biomass Bioenergy* **56**, 479-492 (2013).

34 James, R. Life cycle analysis: power studies compilation report. *National Energy Technology Laboratory, US Department of Energy, Pittsburgh, PA* (2011).

35 Olatunde, A. O. *et al.* Direct calculation of unsteady-state Weymouth equations for Gas volumetric flow rate with different friction factors in horizontal and inclined pipes. (2012).

36 Weymouth, T. R. Problems in natural gas engineering. *Trans. Am. Soc. Mech. Eng.* **34**, 185-231 (1912).

37 Huancong Shi, W. S., Hualong Xu. Catalytic synthesis process of methyl succinic acid. China patent CN1609089A (2004).

38 Nieder-Heitmann, M., Haigh, K. F. & Görgens, J. F. Life cycle assessment and multi-criteria analysis of sugarcane biorefinery scenarios: Finding a sustainable solution for the South African sugar industry. *J. Clean. Prod.* **239**, 118039 (2019).
